# Aggregation of Amyloidogenic Peptide Uperin—Molecular Dynamics Simulations

**DOI:** 10.3390/molecules28104070

**Published:** 2023-05-13

**Authors:** Elena Ermakova, Olga Makshakova, Rauf Kurbanov, Ilya Ibraev, Yuriy Zuev, Igor Sedov

**Affiliations:** 1 Kazan Institute of Biochemistry and Biophysics, FRC Kazan Scientific Center of RAS, Lobachevsky Str., 2/31, Kazan 420111, Russia; 2 Chemical Institute, Kazan Federal University, Kremlevskaya Str., 18, Kazan 420008, Russia

**Keywords:** uperin, molecular dynamics simulations, amyloid peptides, aggregation

## Abstract

Uperin 3.5 is a remarkable natural peptide obtained from the skin of toadlets comprised of 17 amino acids which exhibits both antimicrobial and amyloidogenic properties. Molecular dynamics simulations were performed to study the β-aggregation process of uperin 3.5 as well as two of its mutants, in which the positively charged residues Arg7 and Lys8 have been replaced by alanine. All three peptides rapidly underwent spontaneous aggregation and conformational transition from random coils to beta-rich structures. The simulations reveal that the initial and essential step of the aggregation process involves peptide dimerization and the formation of small beta-sheets. A decrease in positive charge and an increase in the number of hydrophobic residues in the mutant peptides lead to an increase in the rate of their aggregation.

## 1. Introduction

Misfolding of proteins and the formation of amyloid fibrils are associated with a number of incurable neurodegenerative diseases, including Alzheimer’s, Parkinson’s, Creutzfeldt–Jacob’s diseases, and many others [1,2,3]. These disorders arouse the justified interest of scientists. Beta-amyloid, islet amyloid polypeptide, α-synuclein, and prion proteins are all fibril-forming proteins. Many other proteins and peptides also demonstrate amyloidogenic properties. Structural, biophysical, and biological properties of fibrils as well as the mechanism of self-association of amyloidogenic peptides, have been intensively investigated. However, the molecular nature of amyloid peptides’ transformation from monomers to beta-sheet aggregates has not yet been fully uncovered.

Various simulation strategies and especially molecular dynamics simulations are commonly used to assess the mechanisms of aggregation and fibril formation by proteins and peptides. This can be illustrated with a few examples of recent studies. A combination of theory, simulations, experiments, and meta-data analysis were performed to determine the entropic and enthalpic contributions of amyloid fibril elongation [4]. Umbrella sampling, replica-exchange, and conventional molecular dynamics (MD) simulations were used to elucidate the helix–fibril transition pathway of intrinsically disordered α-synuclein [5]. Many simulations were performed for short peptides having 6–8 residues. For example, the GNNQQNY fragment from the yeast prion protein SUP35, hexapeptides from the tau protein, and insulin were used for studying the early stages of amyloid formation [6,7,8]. Multiscale molecular dynamics simulations were applied for the rational design of α-helical peptide that can aggregate into fibrils [9], and to study the aggregation pathways from the initial random distribution of free monomers to the formation of large aggregates [10]. Recently, Jalali et al. analyzed in detail the aggregation process of short model amphipathic peptides, and demonstrated the leading role of hydrophobic interactions in peptide self-assembling [11].

Antimicrobial peptides (AMP) such as protegrins, bacteriocins, and temporins have been described to form typical amyloid-like fibrillar structures, and display distinct characteristics of amyloids, such as increased fluorescence of Thioflavin-T upon binding. A representative of AMP is a family of uperins, which are peptides secreted on the skin of *Uperoleia mjobergii* (Australian toadlet) [12]. They form fibrils with a structure similar to those formed by Aβ-peptides [2,13]. The uperin family peptides have 17–18 residues, carry a small positive charge, and are convenient candidates to study the fibril formation process both in vitro and in silico. Calabrese et al. have shown that uperin fibrils are cytotoxic for model neuronal cells in a similar fashion to those formed by the proteins implicated in neurodegenerative diseases [14]. Amyloid structures formed by uperins were detected by circular dichroism and Thioflavin-T fluorescence [14]. The cryo-EM structure of uperin fibrils was recently published [15]. A combination of experimental and simulation approaches have been used to study the properties of uperin peptides and the process of their aggregation [2,16,17]. It has been shown that the monomeric uperin has a random-coil structure in water, and the fibril association can be initiated upon the addition of salts or buffer to the solution [14,17]. The mutant peptide with Arg7 residue replaced with alanine demonstrates a stronger propensity for amyloidogenesis [2,14]. Another interesting feature of uperins is that, unlike most amyloid peptides, uperins can form not only beta-rich amyloids but also helical cross-α amyloids. Their crystal structure was determined by Salinas et al. [18].

Here, we used uperin peptides as a model to study the early stage of amyloid fibril formation using molecular dynamics simulations. According to the classical model, this process includes nucleation and fibril growth steps. The nucleation process involves the formation of oligomer-like early aggregates [19] with a disordered structure, which subsequently reorganizes into an ordered structure [7]. Our simulations allow us to observe the spontaneous formation of these aggregates by peptides. The effect of point mutations, and the balance of hydrophilic and hydrophobic residues on the rate of peptide aggregation were analyzed. The replacement of positively charged Arg7 or Lys8 residues in wild-type uperin with hydrophobic alanine residues, accompanied with a decrease in positive charges, leads to an increase in the rate of peptide association, which is consistent with the experimental data.

## 2. Results

### 2.1. Formation of Dimers and Beta-Strands Is the First Step of Peptide Aggregation

To investigate the initial stage of fibrillation in detail, we simulated the behavior of wild-type uperin 3.5 (Up) (GVGDLIRKAVSVIKNIV-NH2) and the Up7a (R7A) mutant at a low peptide concentration (7.5 mM), starting with ten unstructured molecules randomly positioned in the water box (trajectories Tr1 and Tr2). Simulation parameters and description of the trajectories can be found in Section 4 Methods.

**Figure 1:**
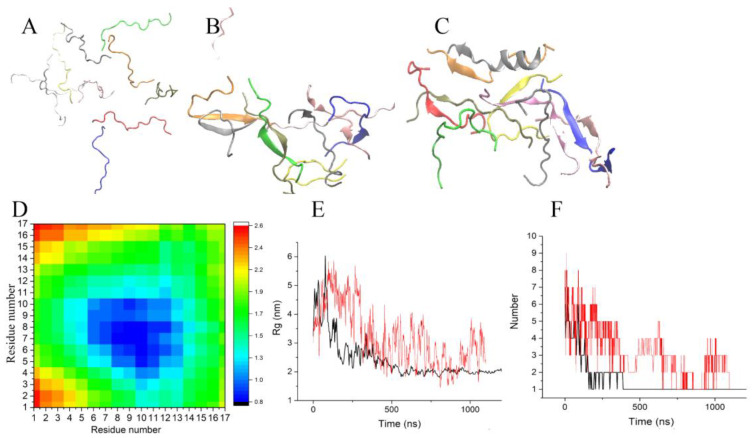
Snapshots of the trajectory Tr2. The peptides are shown in distinct colors. (**A**) The initial configuration of the peptides (t = 0 ns). (**B**) Small cluster formation during the trajectory (snapshot at t = 200 ns). (**C**) The final configuration of ten Up7a peptides (at t = 1 µs). (**D**) The intermolecular residue-residue contact map averaged over the four dimers depicted in Figure 1B. (**E**,**F**) Radius of gyration, number of clusters in the cluster of wild-type uperin (red line) and mutant (black line).

During the MD simulations, we observed the aggregation of the peptides and the subsequent transformation of their structure. As an example, representative snapshots from the simulation of Up7a peptide are shown in Figure 1A–C. They illustrate the spontaneous self-assembly of the peptides.

The initial random configuration of ten peptides is shown in Figure 1A. Spontaneous dimer formation occurred within the first 200 nanoseconds (Figure 1B), while the other peptides were in monomer form. Peptide monomers adopted predominantly coiled conformations. At several instances, monomers could form beta-hairpins or short alpha-helixes, but they were very short-lived. To analyze how the dimers were formed, and which amino acid residues primarily interacted with other peptides, the intermolecular distances between the different residue pairs were calculated. Results were averaged over the first 200 ns and over the four dimers depicted in Figure 1B. Figure 1D shows the intermolecular residue-residue contact map. Hydrophobic residues from Leu5 to Ile13 primarily interacted with other peptides and mainly formed peptide-peptide contacts. Formation of one, two, or three hydrogen bonds between the monomers stabilizes these dimers and promotes beta-strand formation. In the dimers, peptides were associated with each other both in a parallel and antiparallel fashion.

The aggregates grew by merging small clusters and joining the monomers to these formed clusters. For Up7a peptides, the radius of gyration (Rg) of these clusters decreased monotonously with time from 5–6 nm at the beginning of the trajectory to 2 nm during 500 ns and did not change significantly afterward (Figure 1E). Up peptides formed a looser aggregate with Rg equal to 3 nm and aggregated slower than Up7a peptides. Cluster analysis confirmed the association of the peptides. Figure 1F shows the time evolution of the number of clusters. For mutant peptides, the number of clusters decreased to 1–2 showing that all peptides were organized into one aggregate during the first 250–400 ns. For the wild-type uperin, this process took about 1 µs.

At the first stage of aggregation, several small beta-sheets with two, three, or four beta-strands were formed, with some peptides being monomeric and retaining random-coiled conformations. All peptides then became organized into a single cluster, which was quite loose. Peptides interacted with each other weakly and were able to reorient relative to each other.

As seen from the cluster and secondary structure analyses, the formation of dimers and hydrogen bonds between them was necessary for the beta-strand construction. Peptide dimerization and the formation of small beta-sheets is a primary step of the aggregation process. Visual analysis of the trajectory revealed that aggregates grew both by monomer addition to the formed cluster and by the fusion of two small clusters.

On an important note, although the association of peptides occurred relatively fast, the reorganization of clusters into a single regular structure was not observed. 

### 2.2. Influence of a Single Mutation on the Aggregation Properties of Uperins

#### 2.2.1. Aggregation of Wild-Type Uperin

**Figure 2:**
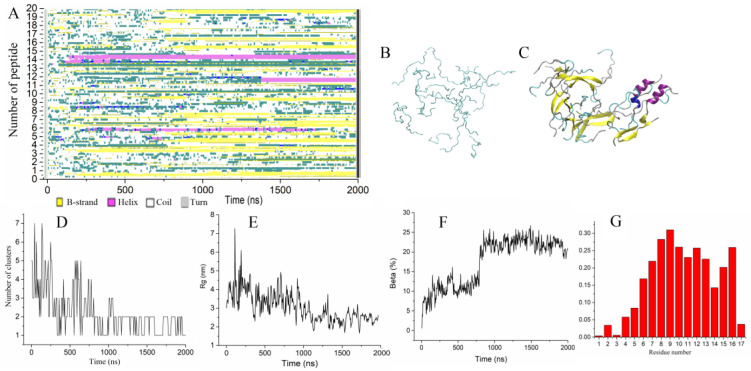
(**A**) Time evolution of the peptide secondary structure. (**B**,**C**) Initial and final configurations of 20 Up peptides. Peptides are shown as cartoons, and β-strands are colored yellow. (**D**–**F**) Number of clusters, radius of gyration, and the fraction of residues forming beta-strands vs. time. (**G**) Probability of residues to form a β-strand.

To accelerate the aggregation process and analyze the effect of the peptide concentration, we doubled the peptide concentration (trajectory Tr3). Figure 2A illustrates the transformation of the secondary structure of the peptides along the trajectory. Figure 2B shows the initial configuration comprising twenty unstructured uperin peptides randomly positioned in the water box at 15 mM concentration. The final configuration of twenty peptides is depicted in Figure 2C. The first elements of the secondary structure began to form after 100 ns of simulation when four peptides constituted into two dimers with short beta-strands. Hydrogen bonds formed between the monomers thereby stabilizing the dimers. Other peptides remained in random coil conformation and grouped into very loose clusters (Figure 2D). This first stage of initial aggregation lasted approximately 800 ns, during which the average radius of gyration of peptides decreased to 3.5 nm (Figure 2E), and a single large cluster was formed as a result. Then, until the end of the simulation, only one peptide periodically joined and left the cluster (Figure 2D).

During the next 1.2 µs, the radius of gyration monotonically decreased to 3 nm (Figure 2E), and the fraction of the beta-strands increased due to the elongation of beta-strands and formation of trimers by attaching monomers to dimers. At the end of the 2 µs simulation, the aggregate consisted of six small beta-sheets randomly oriented relative to each other. Each beta-sheet was formed by three or two peptides. Two peptides also contained short alpha-helices; the others were unstructured (Figure 2C). The proportion of residues involved in the beta-sheets did not exceed 25% (Figure 2F). The profile of the participation of residues in the formation of the beta sheet showed that it mainly involved residues from six to sixteen (Figure 2G).

Even though the formed beta-rich cluster was quite loose, the interactions between the beta-sheets stabilized their relative orientation and prevented them from reorienting. Several peptides were found to be able to participate simultaneously in two beta-sheets, thereby stabilizing their interaction.

#### 2.2.2. Aggregation of Uperin Mutants

To better understand the effects of substituting the positively charged amino acids in uperin with a nonpolar alanine residue on the aggregation process, we performed MD simulations for two mutants, one of which has an Ala residue in the seventh position of Up (Up7a) instead of Arg7, and the other one having alanine in the eighth position (Up8a) instead of Lys8. Similar to the simulation of the wild-type uperin, twenty unstructured mutant molecules were randomly positioned in the water box, and the simulation was performed during 2 µs for Up8a (trajectory Tr4) and during 3 µs for Up7a (trajectory Tr5).

**Figure 3:**
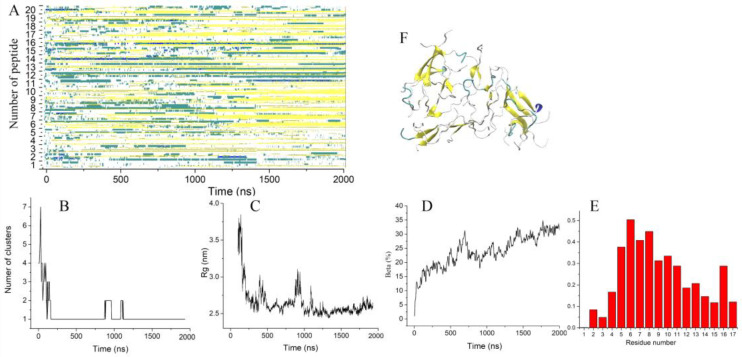
(**A**) Time evolution of the Up8a peptide secondary structure. (**B**–**D**) Number of clusters, radius of gyration, the fraction of residues forming β -strands vs. time. (**E**) Probability of residues to form β-strands. (**F**) The final configuration of 20 peptides.

In comparison to wild-type uperin, Up8a peptides demonstrated more rapid formations of the short β-sheets (Figure 3A). The first dimers appeared after 40–50 ns of simulation. 

All peptides were found to have joined a single loose cluster after 200 ns (Figure 3B). The average radius of gyration of the cluster decreased within 1 µs (Figure 3C) and reached the value of 2.5 nm, which was noticeably less than that of wild-type uperin. The aggregate was stable until the end of the simulation, and the dissociation of the peptides was not observed. The aggregate included all peptides, but most of them were partially disordered. The proportion of residues involved in beta-strands increased with time and reached 32%, which was slightly higher than for wild-type uperin (Figure 3D). At the end of the simulation, the aggregate contained several subdomains consisting of β-sheets that were randomly oriented with respect to each other. Each β-sheet contained from two to five β-strands. Residues from Leu5 to Ser11 and Ile16 demonstrated a higher probability to form β-strands than other residues (Figure 3E,F).

**Figure 4:**
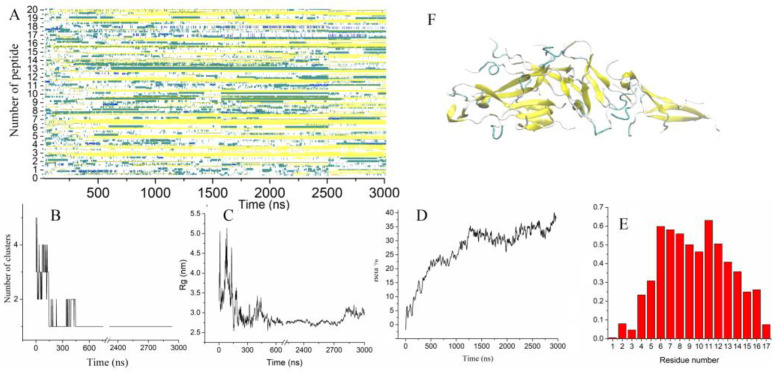
(**A**) Time evolution of the peptide secondary structure. (**B**–**D**) Number of clusters, radius of gyration, the fraction of residues forming β-strands vs. time. (**E**) Probability of residues to form β-strands. (**F**) The final configuration of 20 Up7a peptides.

Similar to Up8a peptides, Up7a mutants formed several tiny beta-sheets consisting of two or three peptides very quickly (Figure 4A). These beta-sheets were organized into small clusters, which became associated into one large cluster at about 150 ns (Figure 4B). From time to time, monomeric peptides either joined the aggregate or dissociated from it. After approximately 400 ns, the cluster no longer dispersed into smaller clusters, and the average radius of gyration of the aggregate did not demonstrate significant fluctuations (Figure 4C). During the first part of the trajectory, the fraction of residues involved in beta–strands increased monotonically with time (Figure 4A,D). In the subsequent part of the trajectory, the reorganization of the associate was observed. The radius of gyration monotonically decreased, showing that the peptides were packed more tightly in the aggregate. The growth of the beta content was achieved by adding monomers, merging pairs of beta-sheets, and lengthening beta-strands. To obtain a more detailed analysis of the reorganization of peptides in the aggregate, this trajectory was prolonged up to 3 µs. Further reorganization of the peptides included in the aggregate occurred very slowly. The fraction of residues forming beta-sheets also grew slowly, reaching 40% by the end of the simulation and showed a tendency to a further increase (Figure 4A,D). Similar to the other trajectories, the central part of the proteins, including residues five to sixteen, had the highest probability to adopt the beta-sheet conformation (Figure 4E). 

**Figure 5:**
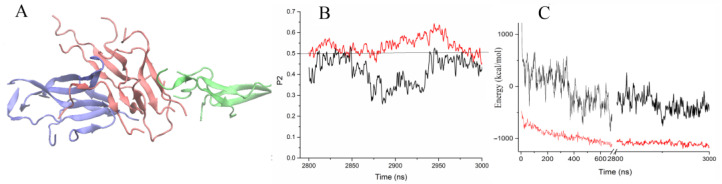
(**A**) Three subdomains formed by 18 peptides. Distinct colors were used for the subdomains that were loosely connected to each other. (**B**) Nematic order parameter P2 for 20 peptides (black line) and for 12 peptides (red line) for the last 200 ns of the trajectory. The horizontal line (at P2 = 0.5) is shown for better visualization. (**C**) Electrostatic (black line) and van der Waals (red line) interaction energy between the peptides.

By the end of the 3 µs trajectory, 18 out of 20 peptides had some portion of a beta-structure. The fraction of residues involved in beta-structures in a single peptide varied from 30 to 70% (Figure 4F). Two peptides were completely unstructured but were also involved in the aggregate. The smallest of the beta-sheets contained two peptides, and the largest one consisted of six peptides. Both parallel and antiparallel orientations of the peptides in the beta-sheets were observed. The final structure was characterized as three subdomains (Figure 5A) loosely connected to each other, while twelve peptides were found to have formed the aggregate core. The subdomains were found to have a stable orientation relative to each other but do not form a single large beta-sheet. Reorganization of the formed subdomains into one aligned ordered form demands much more time.

To characterize the stability of the relative orientation of peptides in the aggregate, the angles between the main principal axis of inertia of each peptide and the main principal axis of the aggregate were calculated for the last 200 ns of the trajectory. These angles varied from 20° to 70°. However, each peptide slightly deviated from their average position with a standard deviation of 13°, showing that the relative orientation of each peptide in subdomains was stable for a long time.

To characterize the alignment of the peptides in the aggregate, we used the nematic order parameter P2, which discriminates between ordered and disordered conformations. P2 values larger than 0.5 indicate that peptides are in an ordered, well-aligned state [20]. Figure 5B shows the nematic order parameter P2 for 20 peptides for the last 200 ns of trajectory, which varied in the range of 0.3–0.5, showing that the cluster was quite loose and subdomain reorganization occurred. At the same time, for 12 peptides, the P2 parameter was about 0.5–0.6 (Figure 5B). This part of peptides forming the aggregate core was found to be partially oriented.

The association of the positively charged peptides in an aqueous solution was controlled mainly by hydrophobic interactions. Wild-type uperin and its variants were found to have a small positive charge. The association of peptides and their reorganization increased the interaction energy between the peptides, including both electrostatic and van der Waals interactions. Figure 5A shows how the energy of interaction between the peptides varied along the trajectory. Unfavorable electrostatic interactions between the peptides at the beginning of the trajectory changed to favorable ones when hydrogen bonds between the monomers were formed. Van der Waals interactions gave a large contribution to the interaction energy and stabilized the aggregates. However, the fast aggregation of the peptides into a single cluster, and strong interactions occurring between the subdomains hindered the reorientation of peptides and slowed down the formation of a regularly ordered structure. The reorientation of these peptides required the rupture of the already formed intermolecular hydrogen bonds, so these interactions were deemed to be weak enough for the peptides to reorient relative to each other.

## 3. Discussion

Molecular dynamics simulations were performed to study the process of aggregation of wild-type uperin 3.5 and two mutants, in which positively charged residues Arg7 and Lys8 were replaced by neutral Ala residues. The spontaneous association of the peptides and conformational transitions from random coil to beta-rich structures were observed for all types of peptides. 

According to the modern nucleation model, the aggregation process shows a two-step behavior: first, the association of monomers leads to stable but disordered oligomers, then the reorganization of disordered oligomers leads to ordered fibril nuclei that start to grow rapidly [7,8]. 

Our calculations showed that the aggregation of uperin peptides was consistent with this model. In this work, we observed the first stage of this process (oligomer formation), and the beginning of the second stage (nuclei formation). 

Analysis of five trajectories revealed some common features of the aggregation process. At the first step, two peptides in an extended conformation formed a dimer and initiated the formation of hydrogen bonds between the peptides and small beta-sheets, which were later associated to form clusters. Larger clusters then grew due to the condensation of the small clusters or by the addition of monomers to the already formed cluster.

The first stage of the associate formation was completed when all the clusters were merged into a single loose aggregate. The time required for it varies and depends on both the peptide concentration and the peptide amino acid sequence. At a low concentration, all Up7a peptides assembled into a single aggregate after 400 ns, while for wild-type uperin approximately 1 µs was required. 

The simulations revealed that a decrease in the positive charge in the mutants Up7a, Up8a resulted in an increase in the rate of peptide association. At higher concentrations, the formation of the aggregates by mutants was observed after 150–200 ns. For the wild-type peptides, it took about 800 ns. Additionally, wild-type uperin peptides demonstrated slightly lower β-sheet content, and its maximum was reached later. Several peptides formed short stable alpha-helices in contrast to the mutants. 

Although all the observed aggregates were beta-rich structures, they were topologically quite diverse. The final aggregate included all peptides, however most of them were partially or fully disordered. The second stage of structural conversion of oligomer aggregates into amyloid-like structures was the slowest stage since it was hindered by the interactions present between the peptides. The growth of the beta structures was achieved by lengthening the beta-strands, or by increasing the number of beta-strands in beta-sheets, but the fraction of residues involved in the beta-structures did not exceed 40% by the end of our simulations, which was found to be insufficient for the formation of regularly ordered structures.

Our results are consistent with the circular dichroism (CD) experiments [14,17] which have shown that the uperin wild-type peptides demonstrated lower β-sheet content, along with a propensity to form aggregates compared to their alanine variants.

## 4. Methods

Uperin 3.5 is a short antimicrobial peptide consisting of 17 amino acids (GVGDLIRKAVSVIKNIV-NH2). The structure of wild-type U3.5 in the α-helical form was obtained from Protein Data Bank (code 6FLT, Salinas). Two uperin mutants were constructed using VMD program [21]. Mutant Up7a contains an alanine residue instead of arginine in the seventh position and Up8a has an alanine residue instead of lysine in the eighth position. Consistent with experimental conditions, wild-type uperin and its mutants were amidated at the C-terminus.

### Molecular Dynamics Simulations

Circular dichroism experiments showed that monomeric uperins were disordered in aqueous solutions [17]. Thus, we used a random coil structure as the initial state in simulations of uperins. This structure was obtained from the α-helical form in an accelerated molecular dynamics (AMD) simulation of a single peptide in a water box [22]. The AMD method uses positive dihedral bias potentials to decrease the energy barriers between different conformers. AMD simulations were conducted for 100 ns. For all the peptides, the helical structure began to be disrupted during the first 10 ns, and full destruction was observed after 25–40 ns. Several different random coil configurations of the peptides from each trajectory were used as the initial peptide structures for subsequent simulations.

All production simulations were performed using conventional molecular dynamics. Ten or twenty peptides in different random coil conformations were distributed uniformly in the water box using the PACKMOL program [23]. K^+^ and Cl^−^ ions were then added to obtain 0.3 M ion strength. Increased concentrations of salts were used to decrease the electrostatic interactions between the peptides and accelerate fibril formation. Simulation parameters are listed in Table 1.

All simulations were performed with the GROMACS 5.0.4 MD package [24]. The CHARMM36m force field was used for all components of the system [25]. Non-bonded interactions were calculated within a cutoff of 1.2 nm for the Lennard–Jones and electrostatic interactions. The periodic boundary conditions were employed for all simulations, and the particle mesh Ewald method was used for long-range electrostatic interactions. The temperature was maintained at 303 K using a Nosé–Hoover temperature coupling method with a time constant of 1 ps. For pressure coupling, a semi-isotropic Parrinello–Rahman method with a time constant of 5 ps and compressibility of 4.5 × 10^−5^ bar^−1^ was used. The pressure was maintained at 1 bar, while the simulation time step was set to 2 fs. Each model was equilibrated for 125 ps [26]. 

The GROMACS and VMD tools were used for the analysis of the system characteristics. The peptide secondary structure was determined with the stride program as implemented by the Timeline plugin in VMD [27]. The orientation of peptides was characterized by the angle between the main principal axis of inertia of each peptide (corresponding to the smallest eigenvalue of the moment of the inertia tensor) and the main principal axis of the aggregate defined using the g_principal function of GROMACS. The GROMACS plugin pairdist was used for the calculation of the distances between the peptide residues in the dimers. The GROMACS plugin clustsize was applied for cluster analysis. Two molecules were deemed to belong to one cluster if the distance between them was less than 3.5 Å. 

The nematic order parameter (P2) of the system characterizes the extent of alignment and the relative orientation of the individual peptides [6,20]. Order parameters were calculated using the wordom program [28]. We defined the molecular vector (**z***_i_*) as the unit vector linking the Ca-atoms of the fifth to thirteenth residues of the *i*-th peptide:

(1) $$P_{2}=\frac{1}{N}\overset{N}{\underset{i=1}{\sum }}\frac{3}{2}{\left(z_{i}\cdot d\right)}^{2}-\frac{1}{2}$$

where **d** is a unit vector that defines the preferred direction of alignment, and *N* is the number of peptide molecules in the simulation box. This helped in distinguishing between the ordered and disordered conformations observed. The perfect alignment of all peptides corresponds to the value of 1 (ordered state), while the value of 0 signifies complete disorder.

## 5. Conclusions

The aggregation of the amyloidogenic peptide Uperin 3.5 and its mutants was studied by molecular dynamics simulations. Calculations showed that all peptides rapidly underwent spontaneous aggregation and a conformational transition from random coils to beta-rich structures. The simulations revealed that the initial and essential step of the aggregation process involved peptide dimerization and the formation of small beta-sheets. A decrease in positive charge and an increase in the number of hydrophobic residues in the mutant peptides lead to an increase in the rate of their aggregation.

## Figures and Tables

**Table 1:** Setup of the simulations.

Trajectory	Peptides	Concentration, mM	Ion Strength, M	Number of Peptides	Number of Water Molecules	Length of Trajectory, μs
Tr1	Up	7.5	0.3	10	38,243	1.1
Tr2	Up7a	7.5	0.3	10	38,310	1.2
Tr3	Up	15	0.3	20	41,108	2
Tr4	Up8a	15	0.3	20	41,274	2
Tr5	Up7a	15	0.3	20	41,300	3

## Data Availability

The data presented in this study are available on request from the corresponding author.
